# Bilateral biliary drainage using 6-mm multi-hole metallic stents in a patient with surgically altered anatomy

**DOI:** 10.1055/a-2944-1468

**Published:** 2026-09-08

**Authors:** Tomihiro Iwata, Koh Kitagawa, Jun-ichi Hanatani, Shohei Asada, Kazuki Watanabe, Ayana Sueki, Hitoshi Yoshiji

**Affiliations:** 1 Department of Gastroenterology 12967 Nara Medical UniversityKashiharaJapan

Performing double-balloon enteroscopy–assisted endoscopic retrograde cholangiopancreatography (DBE-ERCP) for malignant hilar biliary obstruction (MHBO) is challenging in patients with surgically altered anatomy because only a limited number of devices can be used. 1​ ​2​ However, recently, a stent-in-stent method using multi-hole self-expandable metallic stents has been reported to be effective for MHBO drainage. 3​ 4​ ​5​

**Fig. 1:**
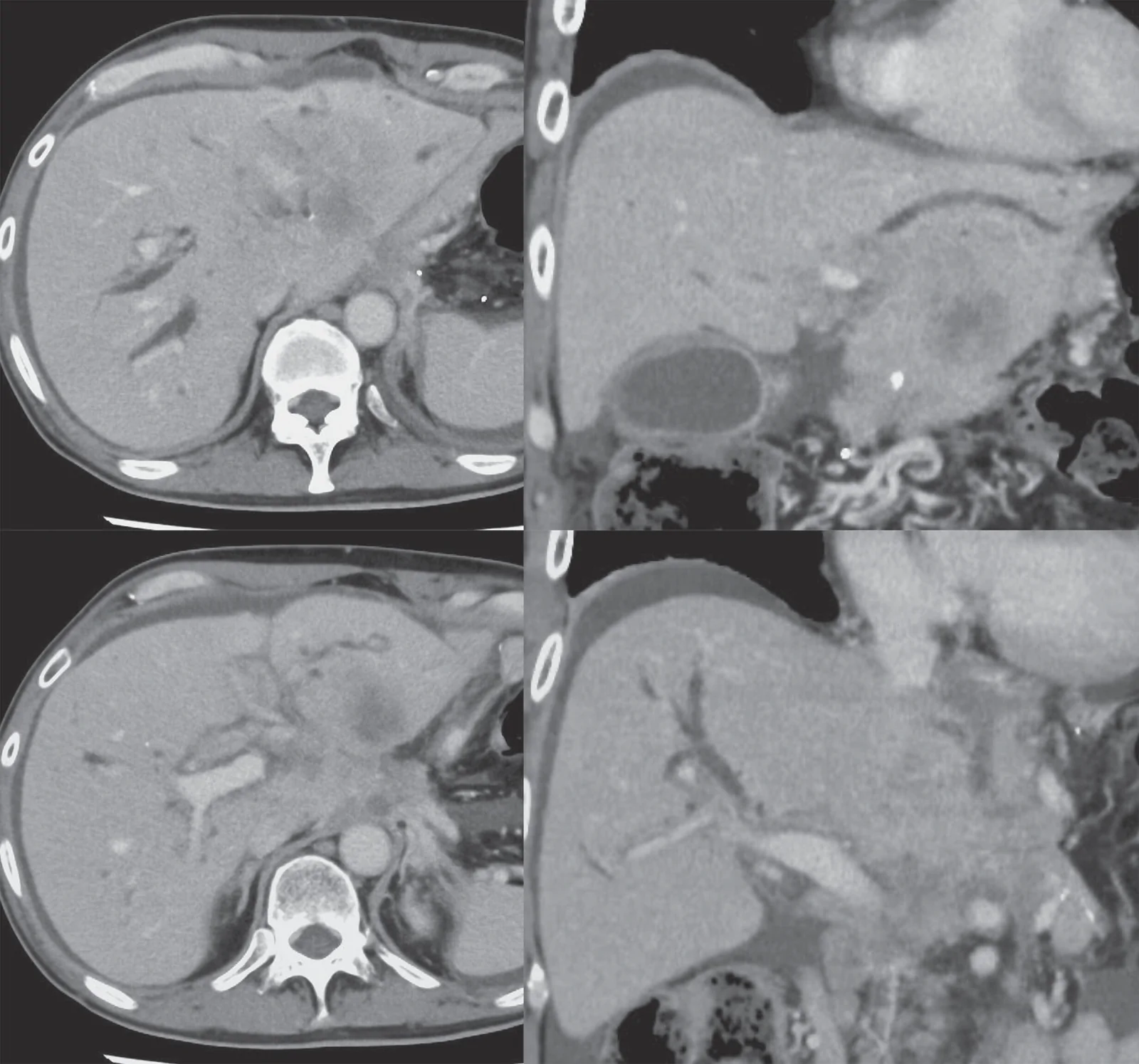
Abdominal contrast-enhanced computed tomography (delayed phase) revealed multiple liver metastasis and hilar biliary obstruction resulting from gastric cancer recurrence. The intrahepatic bile ducts also appeared to be dilated.

**Fig. 2:**
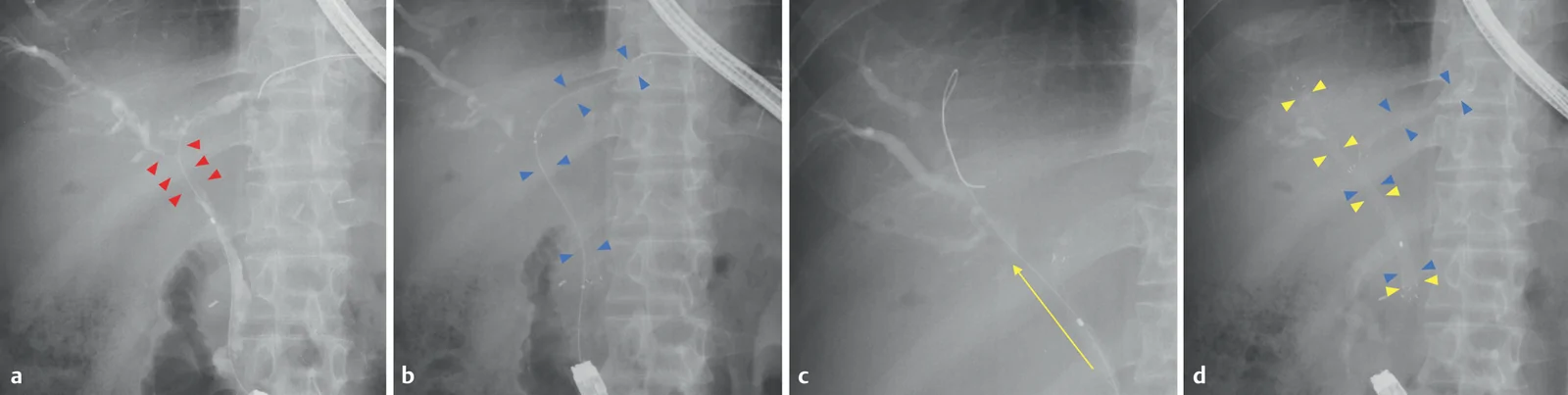
Fluoroscopic images during double-balloon enteroscopy–assisted endoscopic retrograde cholangiopancreatography. ( **a** ) Cholangiography revealed bilateral hepatic duct strictures (red arrowheads). ( **b** ) First, a multi-hole self-expandable metallic stent (6 mm in diameter and 100 mm in length) was placed in the left hepatic duct (blue arrowheads). ( **c** ) The guidewire that had been left in place was pulled back and then advanced into the right hepatic duct through the side-hole of the initially placed multi-hole metallic stent (yellow arrow). ( **d** ) The second multi-hole metallic stent (6 mm×100 mm) was successfully placed in the right hepatic duct (yellow arrowheads) using the stent-in-stent technique without requiring any dilation devices.

**Fig. 3:**
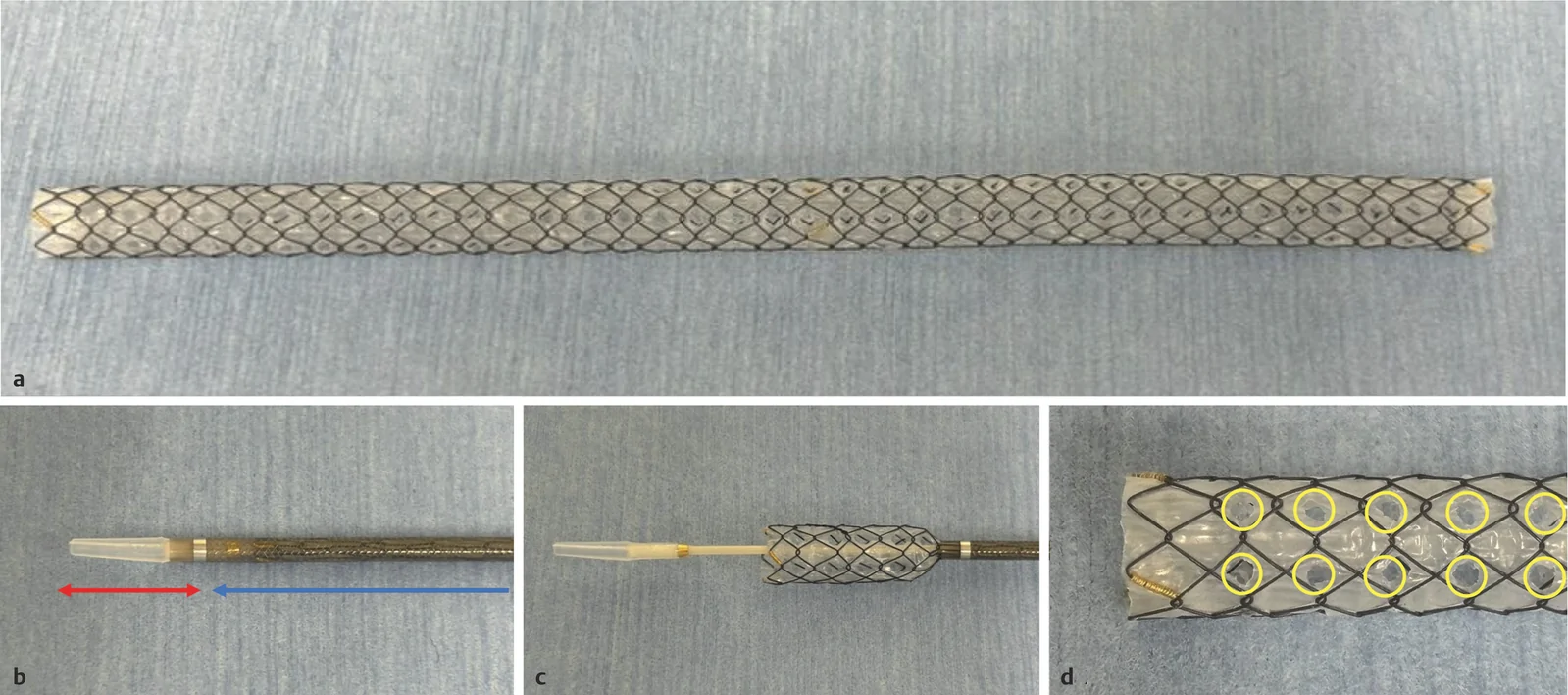
Design of the novel slim multi-hole self-expandable metallic stent. ( **a** ) An overview of the stent. ( **b** ) The diameter of the delivery system’s outer sheath measures 5.9 Fr (blue arrow). Furthermore, the catheter tip is tapered, designed for use with 0.025-inch guidewires, providing excellent penetration capabilities (red arrow). The total length of the delivery catheter is 1,800 mm. ( **c** ) The stent was deployed from the outer sheath. ( **d** ) Six rows of 1.5-mm holes are arranged along the long axis of the stent (yellow circles).

**Fig. 4:**
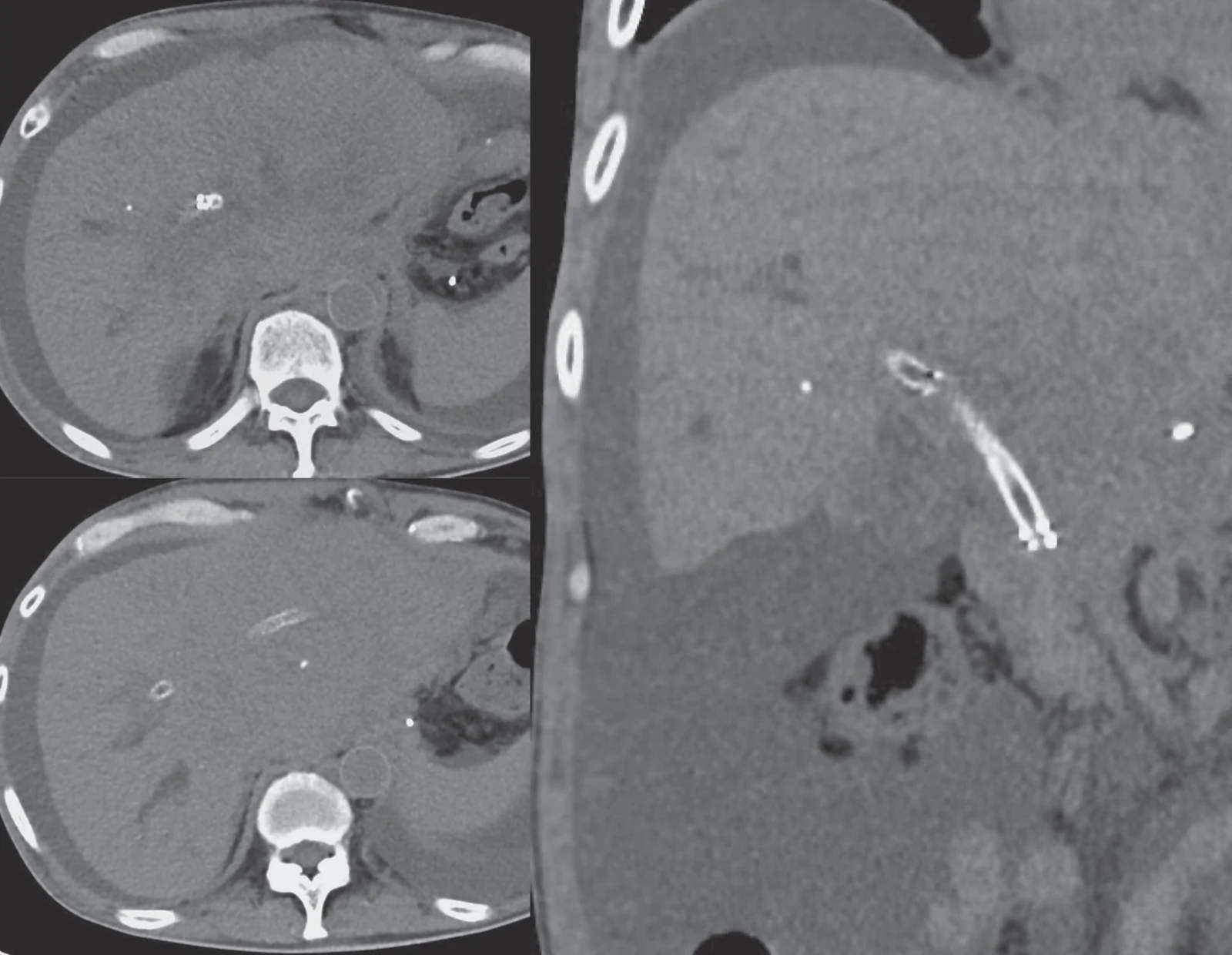
A follow-up abdominal computed tomography scan. The dilation of the intrahepatic bile ducts had improved, with no liver abscesses observed.

A 60-year-old man presented with MHBO secondary to gastric cancer recurrence after total gastrectomy (Roux-en-Y reconstruction; Fig. 1 ). Accordingly, DBE-ERCP was performed ( Fig. 2 , Video 1 ). A 0.025-inch guidewire was first placed in the left hepatic duct. Subsequently, a novel multi-hole metallic stent (6 mm in diameter and 100 mm in length) was deployed ( Fig. 3 ). The stent delivery catheter had a diameter of 5.9 Fr and a total length of 1,800 mm. Subsequently, the guidewire was advanced into the right hepatic duct through the side-hole of the initially placed stent. Using the stent-in-stent technique, a second multi-hole metallic stent (6 mm×100 mm) was deployed. Consequently, jaundice and cholangitis resolved without adverse events ( Fig. 4 ).

**Video 1** Bilateral biliary drainage using 6-mm multi-hole metallic stents during double-balloon enteroscopy–assisted endoscopic retrograde cholangiopancreatography.


This report may be the first to present biliary drainage through the stent-in-stent method using 6-mm multi-hole metallic stents via DBE-ERCP. In biliary drainage for patients with MHBO, draining at least 50% of the total liver volume is desirable. However, multiple stent placement during DBE-ERCP is challenging. The 6-mm multi-hole metallic stent features a slim delivery system, allowing the two multi-hole metallic stents to be easily inserted and deployed without requiring any dilation devices, even during DBE-ERCP. Furthermore, using fully covered metallic stents raises concerns regarding cholangitis resulting from lateral biliary branch obstruction; nevertheless, the multi-hole metallic stent has multiple side holes, which may help prevent such obstruction.

In conclusion, the stent-in-stent method using the novel 6-mm multi-hole metallic stent may be an effective option in patients with MHBO, even during DBE-ERCP.
